# Reliability and validity of the Chinese version of Nighttime Collaboration Difficulties between Nurses and Physicians for Nurses (NCDNP-N) scale

**DOI:** 10.7717/peerj.21097

**Published:** 2026-05-20

**Authors:** Ying Zhang, Di Jiang, Longjiao Wei, Lin Chen, Lianghai Cao, Mengjie Liu

**Affiliations:** Chengdu University of Traditional Chinese Medicine, Affiliated Hospital of Integrated Traditional and Western Medicine, Chengdu, Sichuan, China

**Keywords:** Nurse, Physicians, Nighttime, Collaboration, Reliability, Validity, Scale

## Abstract

**Background:**

The cooperation between nurses and physicians at night is a vital influencing factor of patient treatment and patient safety. Effective communication and collaboration could respond effectively to changes in the patient’s condition at night. There is currently a lack of scale to assess the difficulties in collaboration between nurses and physicians during the night shift. While the scale of Nighttime Collaboration Difficulties between Nurses and Physicians for Nurses (NCDNP-N) has been developed in Japan. However, currently China still lacks a scale for assessing the degree of difficulty in collaboration.

**Objectives:**

To evaluate the reliability and validity of the Chinese version of NCDNP-N scale among nurses in the context of Chinese clinical nursing.

**Methods:**

The translated scale’s reliability was assessed by means of Cronbach’s α coefficient, split-half reliability, and test-retest reliability. Exploratory factor analysis (EFA) and confirmatory factor analysis (CFA) were employed to assess structural validity. The data was analyzed using SPSS 22.0 and Amos 24.0.

**Results:**

This was a cross-sectional study conducted among 483 clinical nurses in China. The Cronbach’s α coefficient of the Chinese version of NCDNP-N scale was 0.922, the Cronbach’s α coefficients of the three dimensions were 0.899, 0.960 and 0.954, respectively. The split-half reliability was 0.874, and the test-retest reliability was 0.886. The cumulative variance contribution rate of the three common factors extracted by exploratory factor analysis with eigenvalues greater than 1 is 86.284%. The results of the CFA showed that the chi-square to degrees of freedom ratio (CMIN/DF) was 1.484, the incremental fit index (IFI) was 0.993 the comparative fit index (CFI) was 0.993, and the goodness-of-fit index (GFI) was 0.963, supporting the efficacy of the scale in assessing the difficulties of cooperation between nurses and physicians of nighttime.

**Conclusion:**

The Chinese version of the NCDNP-N scale demonstrated favourable reliability and validity among nurses, making it a suitable tool for measuring the level of nighttime collaboration difficulties between nurses and physicians for nurses.

## Background

Nurses and physicians play a crucial role in collaboratively ensuring patient safety and preserving patients’ physical and mental well-being (Granel et al., 2020; Lopes et al., 2024). While the medical team functions as a cohesive unit in patient care, each member has distinct roles and responsibilities (Al-Bustanji et al., 2024). Nurses are responsible for patient care, monitoring patients’ conditions, and promptly reporting any changes to physicians (Hart et al., 2016; Jensen, Skår & Tveit, 2018). Meanwhile, physicians assume a central leadership role in addressing changes in patients’ conditions, maintaining situational awareness, and providing timely interventions (DiazGranados et al., 2018). Medical collaboration is a process where physicians and nurses, based on principles of equality, autonomy, mutual respect, and trust, make joint decisions and solve problems through effective communication, diligently fulfilling their respective duties to provide optimal healthcare services to patients (Bridges, 2014). Effective collaboration between nurses and physicians is essential to jointly address changes in a patient’s condition (Johnston et al., 2015).

Previous studies have shown that nighttime is an independent high-risk period for clinical deterioration; delays in physician-nurse collaboration increase the risk of unplanned ICU admission and mortality, and this delay effect is significantly amplified during night shifts (Chen et al., 2015; Fernando et al., 2018). When conducting a retrospective study of the night shift communication records at Stanford hospital in 2014, the researchers collected a total of 1,820 pages of relevant records and selected a 10% sample for in-depth comparative analysis. The results showed that urgent calls accounted for 62.1%, and non-urgent calls accounted for 27.7%, this indicated that nurses communicate most frequently with doctors at night, and communication with urgent content is delivered more frequently than non-urgent messages. It also highlights that patients are more likely to experience abnormal vital signs or requests for evaluation during the night (Sun et al., 2018). However, due to the lack of human resources at night and the absence of the patient’s attending physician, nurses may be forced to work with the on-duty physician who is unfamiliar with the details of the patient’s case, when the physician is unwilling to make decisions and take calls, this is one of the challenges that nurses face when dealing with changes in patients’ conditions (Bingham et al., 2020). Studies of medical communication showed that the effective communication and collaboration between nurses and physicians may be hindered by factors such as insufficient staffing at night, ineffective communication, cognitive differences between professions, psychological stress or emotional fluctuations (Ede et al., 2021; Li et al., 2020). The difficulties and obstacles in the cooperation between nurses and physicians at night will affect the timeliness of patient treatment and even endanger patient safety (Hotta, Ashida & Tanaka, 2024b). Among the factors affecting patient safety, communication barriers have become one of the main reasons for more than 60% of alarm incidents (Riesenberg, Leitzsch & Little, 2009). Night shift communication is a crucial link in the transmission of information between physicians and nurses. It plays an extremely important role in medical work and has some distinct characteristics.

To assess nurse and physicians cooperation, researchers have developed various tools (Hojat & Herman, 1985; Xu, Li & Johnston, 2013). These scales measure abstract nurse-physician cooperation or attitudes and cannot assess specific concepts that reflect the clinical situation of night shifts. Because of their different roles in patient care, nurses and physicians may have different perspectives on the difficulties and factors associated with night collaboration. Effective communication between nurses and physicians continues to be a challenge due to differences in training and ingrained workplace cultures (Chua et al., 2020; Tan, Zhou & Kelly, 2017). Randomized controlled trials have been conducted regarding team communication and attitudes towards team collaboration training, but assessing the difficulties in collaboration between nurses and physicians is a prerequisite for targeted training (Liaw et al., 2020). A previous study on the collaboration between nurses and physicians revealed that heavy clinical workload, organizational constraints, and different power relationships hindered effective communication and collaboration between them (Tang et al., 2018). Currently, there are significant barriers to night shift collaboration and communication between nurses and physicians in China. These barriers stem from multiple ineffective states in communication, psychological pressure in communicating with physicians, cognitive differences, and emotional fluctuations (Li et al., 2020). Despite the fact that literature has identified an issue with night-time physician-nurse communication, the research of specific night-time physician-nurse cooperation remains unexplored. As a result, night shift physician-nurse collaboration has unique characteristics and is a clinical topic requiring future investigation in China. A scale to assess nighttime cooperation difficulties from the perspective of nurses and physicians is necessary.

Hotta, Ashida & Tanaka (2024a) has developed the NCDNP-N scale to effectively assess the difficulties experienced by nurses in working with physicians to cope with deterioration during night shifts, and the scale was applied through the self—report form of nurses. All the factors related to the developed scale were analyzed, including background characteristics, night shift practices, and facility characteristics. The purpose of this scale is to identify the obstacles of cooperation between nurses and physicians in night shift from the perspective of nurses and improve the relationship between nurses and physicians. The scale was found to have high reliability and validity in a specific context in Japan, which further proves its value as an effective tool for assessing the difficulties in nighttime collaboration between nurses and physicians.

At present, most of the existing scales in China focus on the assessment of the level of medical communication (Al-Hamdan, Banerjee & Manojlovich, 2018; Ushiro, 2009), and there is no universal scale that can measure the difficulty of cooperation between nurses and physicians during night shifts. Introducing the NCDNP-N scale within the context of Chinese medical holds significant potential applications. Given the importance of collaboration between night shift healthcare workers for patient safety and clinical outcomes, there is an urgent need to introduce the NCDNP-N scale to assess the cooperation between night shift nurses and physicians. Thus, the purpose of this study is to examine the NCDNP-N scale’s validity and reliability for Chinese nurses.

## Methods

### Design and sample

This study was conducted in January 2025 among clinical nurses in three secondary hospitals and five tertiary hospitals across the provinces of Sichuan, Guizhou, and Anhui in China. The sample source simultaneously covers hospital-level and regional-economic dimensions, making it highly isomorphic with mainstream Chinese healthcare settings and thus demonstrating strong external representativeness. Data collection was conducted using a cross-sectional design in conjunction with a convenience sampling method. To be eligible, registered nurses who work in frontline care positions and regularly undertake night shifts. Nurses undergoing training, further education, or internships, as well as those on leave for over three months, were excluded from the study. The research followed the criterion proposed by Kendall (Li et al., 2024), the sample size should be at least five to ten times the number of items (Kong et al., 2023), as the scale used in this study contained 10 items, we calculated as ten times the number of projects, and considering a 20% rate of invalid questionnaires, the minimum number of valid samples to be included should be 120. In the end, 483 valid samples were included in this study. Before the study began, all participants had provided informed consent and signed the informed consent form, agreeing to participate in this study. Written informed consent was obtained from all participants.

### Instrument

The NCDNP-N scale (Hotta, Ashida & Tanaka, 2024a) was validated among 405 nurses working rotating night shifts in 33 acute-care hospitals in Japan. It is an effective tool for assessing the degree of difficulty in night-time collaboration between nurses and physicians. It consists of 10 items divided into three dimensions: barriers to reporting during night shifts (four items), dissatisfaction with the physicians’ behavior (three items), burden of working with night-shift physicians (three items). The Likert 5-point rating scale was employed, ranging from “never experienced” to “always experienced”. The scale’s total score spans from 0 to 40 points, with higher scores indicating a greater degree of collaboration difficulty between nurses and physicians. The original scale’s Cronbach’s α coefficient is 0.89, and the subscales’ Cronbach’s α coefficients range from 0.76 to 0.82, which suggests that their internal consistency is within an acceptable range. The authors have permission to use this instrument from the copyright holders.

## Procedures

### Translation procedure

After obtaining the authors’ permission, two translators followed the key steps of the modified Brislin’s translation process to translate the original NCDNP-N into Chinese Mandarin, ensuring the scale’s accuracy and cultural adaptability (Wang et al., 2022a; Wang et al., 2022b). The translation process consisted of three steps.

Step 1, forward translation: two bilingual nursing scholars (MSc & PhD) independently produced Chinese versions, one of them is an author of this paper; discrepancies were resolved by the research team.

Step 2, back-translation: a China-born nursing PhD with ≥2 years in an English-speaking country and a university medical English teacher back-translated the merged version; after reconciliation it was sent to the original author for approval.

Step 3, according to the cross-cultural adaptation guidelines for scales, a comparison was made between the original scale and the back-translated English version (Guillemin, Bombardier & Beaton, 1993; Sousa & Rojjanasrirat, 2011), a total of 12 nursing experts from Sichuan, Guizhou, and Hubei provinces were invited to conduct a cultural adaptation of the translated scale and participate in two rounds of expert consultations. The experts evaluated each of the 10 items of the Chinese Mandarin scale in terms of item context, linguistic expression, and applicability, and assessed the content validity of the scale. All 12 experts hold at least a master’s degree, with 10 possessing deputy senior titles above and two serving as supervisor nurses. Each expert has over 10 years of experience in clinical nursing or nursing management. Based on the opinions of translators and experts, the dimensions and items of the scale were modified as follows. Item 2 (The rules and procedures are confusing and difficult) did not conform to our clinical culture, and was replaced with “work systems and procedures are confusing and troublesome.” Item 7 (I feel anxious about the night physician’s response to patients) was easier to understand by replacing it with “I feel anxious about the night-shift physician’s response and disposition to the patient”. Item 9 (I find it difficult to communicate with night-shift physicians because of their personality and the lack of a usual relationship) was ambiguous and replaced with “I find it difficult to communicate with night-shift physicians because of their personality and the lack of daily interaction”. The research team extensively discussed the dimensions and agreed that these modifications were consistent with the Chinese cultural context and expression. Moreover, the process of translating and culturally adapting scales often involves such modifications (Luo et al., 2023; Wang et al., 2023), thereby enhancing the understanding and acceptance of each scale item.

Finally, a pilot experiment was conducted with 30 clinical nurses recruited from a tertiary medical institution in Chengdu, China. Upon completion of the scale, all participants indicated that the items were clearly articulated and easy to comprehend. The time required to complete the scale was 3–5 min, and no changes were made to the items. The feedback from these pilot participants provided further evidence of the clarity and usability of the translated scale.

### Data collection procedure

This study adopted a convenience sampling design to collect data. To conduct the survey, an online platform called Wenjuanxing (http://www.wjx.cn) was utilized. Prior to conducting the survey, we obtained the consent and cooperation of the nursing department at the surveyed hospital. The initial page of the questionnaire includes detailed instructions that elucidate the significance, objectives, and precautions of this study. This survey adheres to the principles of anonymity and confidentiality, with all data being utilized exclusively for this research.

### Data analysis

During the data analysis process, all abnormal questionnaires that showed obvious patterns or contain perplexing logical contradictions, such as completely consistent option selections or conflicting responses, were excluded. Two weeks later, 30 nurses were selected for a retest to assess the retest reliability of the scale (Xu et al., 2025). Descriptive analysis was conducted using mean ± standard deviation, frequency, and percentage. The scale was subjected to item analysis using the critical ratio method and Pearson correlation coefficient method. Reliability was assessed using Cronbach’s alpha coefficient, split-half reliability, and test-retest reliability. Validity was evaluated through content validity index (CVI) and structural validity. The final participants were randomly assigned to two groups at a 1:1 ratio: 241 participants were assigned to exploratory factor analysis (EFA), while 242 participants were assigned to confirmatory factor analysis (CFA) (McDonald & Ho, 2002). Confirmatory factor analysis was performed using AMOS software (IBM, Armonk, New York, USA) to examine the model’s goodness-of-fit indices. As a professional structural equation modeling software, AMOS offers significant advantages in confirmatory factor analysis by simultaneously handling measurement errors and latent variable relationships. The software also provides comprehensive model-fit indices that allow for a thorough evaluation of how well the theoretical model matches the empirical data. EFA was performed in SPSS (IBM) using principal component analysis and direct oblique rotation with maximum variance. The statistical analysis was conducted using SPSS version 22.0 (IBM) and AMOS version 24.0 (IBM).

### Ethics approval

The study protocol was approved by the Medical Ethics Review Committee of Chengdu Integrated TCM&Western Medicine Hospital (approval number: 2025-YNYJ-008). And all participants provided informed consent to take part in the study.

## Patient and Public Involvement

There were no patients or members of the public involved in the research.

## Results

### Demographic information

A total of 512 questionnaires were collected, and 483 were valid, with an effective recovery rate of 94.3%. The features of the participants in the EFA group (*n* = 241) and the CFA group (*n* = 242) are encapsulated in Table 1.

**Table 1:** Features of the participants.

Variables		EFA (*n* = 241)	CFA (n=242)
Sex	Male	29 (12.0%)	18 (7.4%)
	Female	212 (88.0%)	224 (92.6%)
Age (years)	<25	5 (2.1%)	14 (5.8%)
	25 ∼30	116 (48.1%)	119 (49.2%)
	31 ∼40	101 (41.9%)	101 (41.7%)
	41∼51	19 (7.9%)	8 (3.3%)
Work years	1 ∼5	77 (32.0%)	91 (37.6%)
	6 ∼10	93 (38.6%)	103 (42.6%)
	11∼20	56 (23.2%)	43 (17.8%)
	≥20	15 (6.2%)	5 (2.1%)
Education level	College	28 (11.6%)	20 (8.3%)
	Undergraduate	208 (86.3%)	219 (90.5%)
	Postgraduate	5 (2.1%)	3 (1.2%)
Job title	Nurse	33 (13.7%)	32 (13.2%)
	Senior Nurse	117 (48.6%)	138 (57.0%)
	Supervisor Nurse	83 (34.4%)	66 (27.3%)
	Co-Chief Nurse and above	8 (3.3%)	6 (2.5%)
Number of night shifts per month	1∼5	116 (48.1%)	122 (50.4%)
	6∼9	58 (24.1%)	88 (36.4%)
	≥10	67 (27.8%)	32 (13.2%)
Hospital grade	Secondary hospital	63 (26.1%)	68 (28.1%)
	Tertiary hospital	178 (73.9%)	174 (71.9%)
Shift pattern	Two-shift system	47 (19.5%)	71 (29.3%)
	Three-shift system	167 (69.3%)	142 (58.7%)
	Others	27 (11.2%)	29 (12.0%)
Department	Medical ward	37 (15.4%)	37 (15.3%)
	Surgical ward	55 (22.8%)	80 (33.1%)
	ICU	28 (11.6%)	53 (21.9%)
	Emergency	37 (15.4%)	2 (0.8%)
	Department of pediatrics	16 (6.6%)	11 (4.5%)
	Gynecology and obstetrics	27 (11.2%)	27 (11.2%)
	Others	41 (17.0%)	32 (13.2%)

**Notes.**

EFA exploratory factor analysis CFA confirmatory factor analysis ICU Intensive Care Unit

### Item analysis

In this investigation, Pearson correlation analysis was employed to assess the relationship between the items and the total score. A statistically significant strong correlation was identified (*p* < 0.001), with correlation coefficients varying from 0.696 to 0.870. Each item exhibited a positive correlation with the total score, indicating a high degree of correlation. Additionally, the critical ratio for the 10 items in this study ranged from 13.517∼29.956 Table 2.

**Table 2:** Critical ratios of Nighttime Collaboration Difficulties between Nurses and Physicians for Nurses (NCDNP-N) scale, item-total correlation coefficients.

Item	Critical ratio	Correlation item total score	*p*
A1	14.907	0.713	<0.01
A2	13.987	0.723	<0.01
A3	13.517	0.696	<0.01
A4	13.600	0.713	<0.01
A5	29.956	0.821	<0.01
A6	29.902	0.870	<0.01
A7	25.908	0.849	<0.01
A8	17.846	0.778	<0.01
A9	19.223	0.792	<0.01
A10	20.023	0.807	<0.01

### Validity analysis

#### Content validity

The Chinese version of the NCDNP-N was evaluated by a panel comprising 12 experts. The results of this evaluation indicated that the item level content validity index (I-CVI) scores ranged from 0.83 to 1.00, while the scale-level content validity index (S-CVI) value was 0.98.

#### Construct validity

##### Exploratory factor analysis.

The 483 questionnaires were randomly allocated into two groups in a 1:1 ratio for subsequent analysis. 241 questionnaires were utilized for exploratory factor analysis (EFA). The EFA yielded a Kaiser-Meyer-Olkin (KMO) measure of 0.879 and a significant Bartlett’s test of sphericity value of 2,450.606 (*P* < 0.001), which suggests a robust intercorrelation among the variables and their appropriateness for factor analysis (Liu et al., 2022). By employing principal component analysis and direct oblique rotation (Schreiber, 2021), three factors with eigenvalues exceeding 1 were identified, accounting for a cumulative variance of 86.284%. The rotated component matrix showed that all item loadings were higher than 0.4, spanning from 0.754 to 0.906. See details in Table 3.

**Table 3:** Factor loading of each item in NCDNP-N scale (*n* = 241).

Item	F1	F2	F3
A2 It is difficult for me to contact physicians during the night shift because the work system and procedures are confusing and cumbersome.	0.844		
A1 It bothers me when I can’t immediately reach a physician during night shifts.	0.837		
A3 I am hesitant to contact a physician during night shifts out of consideration.	0.828		
A4 I have difficulty reporting changes in a patient’s condition to night-shift physicians to ensure that they are aware of the situation.	0.754		
A5 When I call for night-shift physicians, it takes them a long time to come to the ward or bedside.		0.906	
A6 I have trouble because the night-shift physician’ response to the call is reluctant.		0.887	
A7 I feel anxious about the night-shift physician’s response and disposition to patients.		0.880	
A9 I find it difficult to communicate with night-shift physicians because of their personality and the lack of daily interaction.			0.900
A10 I have difficulty interacting with night-shift physicians due to their unreasonable attitudes or reactions.			0.878
A8 I strongly feel it is a burden to assist the night-shift physician in responding to patients at night.			0.869
Eigenvalue	6.109	1.320	1.199
Cumulative variance contribution rate (%)	30.814	58.617	86.284

**Figure 1:**
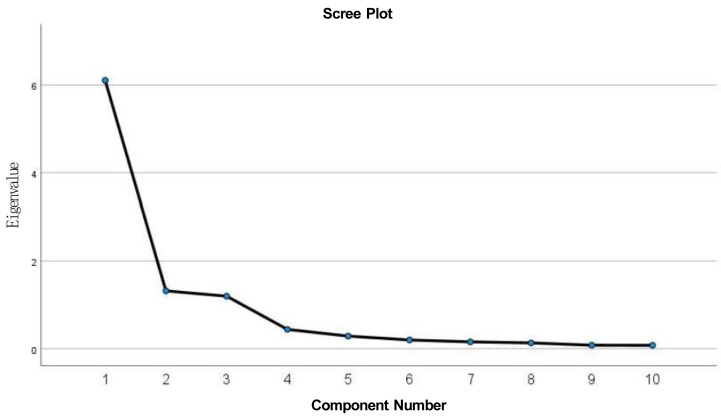
Scree plot of the three-factor model.

The analysis indicated that all 10 items exhibited loadings above 0.4, which implies that all items should be retained. The scree plot (see Fig. 1) displayed a flattening trend following the third factor, indicating the absence of additional significant factors. Thus, retaining three factors was deemed appropriate.

##### Confirmatory factor analysis.

**Figure 2:**
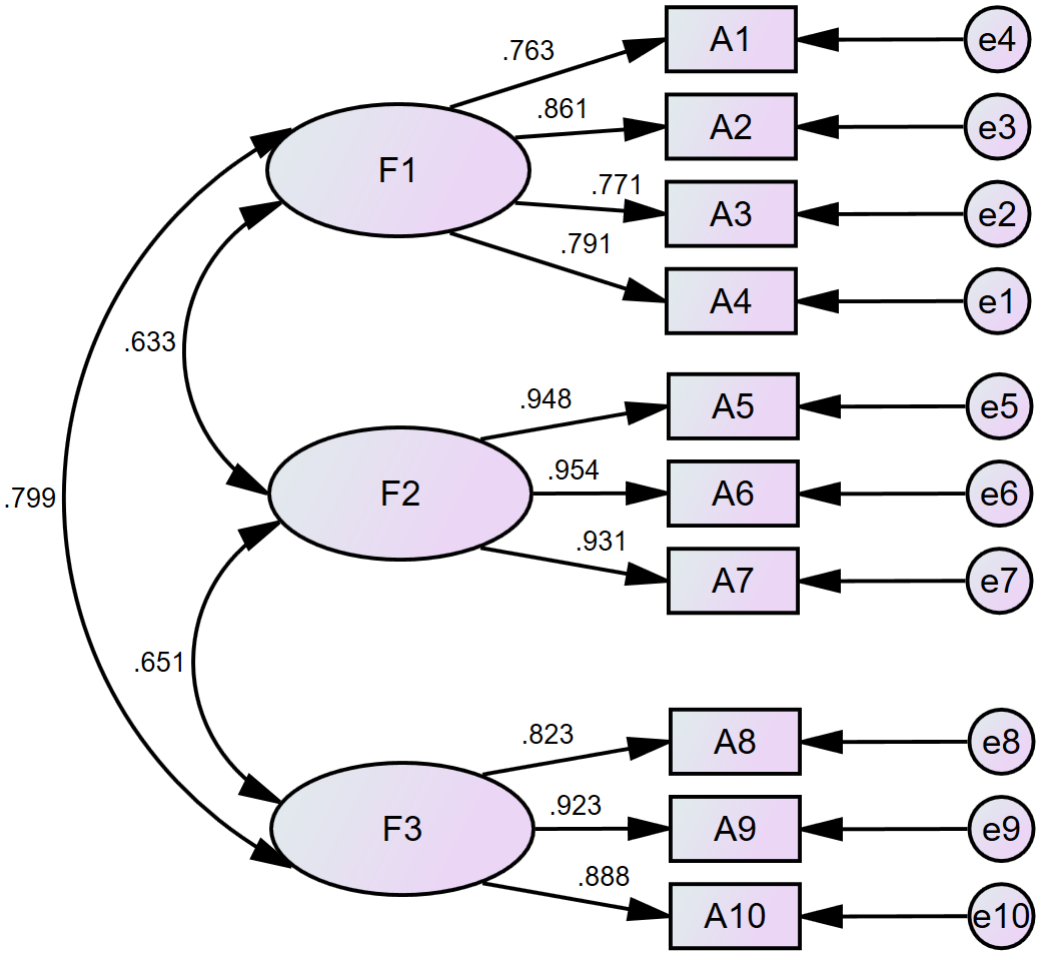
Standardized three-factor structural model of NCDNP-N.

Maximum likelihood method was used to conduct confirmatory factor analysis on the data. The model fit indices were as follows: chi-square to degrees of freedom ratio (CMIN/DF) = 1.484, comparative fit index (CFI) = 0.993, goodness-of-fit index (GFI) = 0.963, incremental fit index (IFI) = 0.993, Tucker-Lewis index (TLI) = 0.990, root mean square residual (RMR) = 0.022, root mean square error of approximation (RMSEA) = 0.045, all the indices met the recommended standards, indicating that the model constructed using AMOS 24.0 software had a good fit (Hu & Bentler, 1999). The results of Confirmatory factor analysis are performed in Fig. 2.

#### Convergent validity and discriminant validity

The composite reliability (CR) values of the three dimensions of the scale were 0.875, 0.961, and 0.911, respectively, all greater than 0.7, the average variance extracted (AVE) values were 0.636, 0.892, and 0.773, respectively, all greater than 0.45, indicating the convergent validity of the scale was acceptable. The correlation coefficients of all dimensions ranged from 0.633 to 0.799 (all *p* < 0.01), and the square root of AVE value was greater than the corresponding correlation coefficients, indicating that the scale had good discriminant validity.

### Reliability analysis

The Cronbach’s α coefficients value for the Chinese version of NCDNP-N scale was found to be 0.922, the Cronbach’s α coefficients of the three dimensions were 0.899, 0.960 and 0.954, respectively, suggesting good reliability. And split-half reliability to be 0.874, indicating high internal consistency. To evaluate the test-retest reliability, the scale was re-administered to 30 nurses following a two-week interval, yielding a reliability coefficient of 0.886. This result demonstrates that the scale exhibits satisfactory stability and consistency across different time points.

## Discussion

The study findings indicated that the Chinese version of the NCDNP-N scale is a valid and reliable instrument for evaluating the extent of nighttime collaboration difficulties between nurses and physicians among Chinese nurses. The original scale was adapted and translated in accordance with Brislin translation principles (Brislin, 1970). Additionally, several nursing experts participated in this process to ensure that the translation aligns with Chinese idiomatic expressions and linguistic patterns. Following translation and cross-cultural adaptation based on international standards, the Chinese version of the NCDNP-N scale demonstrated robust internal consistency and appropriate applicability in the context of China.

The S-CVI of the Chinese version of NCDNP-N scale was 0.98 > 0.900, additionally, the I-CVI was 0.83–1.00 > 0.800. This indicates that the content validity of the scale is good, maintaining strong relevance and accuracy in its content, and is able to better reflect the constructs being measured. The correlation coefficients between each item and the total score of the scale ranged from 0.696 to 0.870 (*p* < 0.001), all of which were greater than 0.4, indicating that the scale had high internal consistency. Exploratory factor analysis extracted three factors from the translated scale, with the composition and allocation of items consistent with the original version, indicating that the core conceptual dimensions of the scale were effectively preserved. The results of the confirmatory factor analysis revealed that the CMIN/DF was 1.484, with CFI, GFI, IFI, and TLI all exceeding 0.9. Additionally, RMR was 0.022 and RMSEA was 0.045. These fit indices collectively met the established criteria, indicating satisfactory structural validity for the Chinese version of the NCDNP-N scale. Furthermore, the results demonstrated that the CR values for each dimension exceeded 0.7, AVE values surpassed 0.45, and the square roots of AVE were greater than the correlation coefficients between specific factors of the scale. It confirms that the items in the scale correspond well with their respective dimensions while maintaining distinct differentiation among the dimensions, making them more aligned with Chinese nursing culture and language practices.

Reliability, which reflects the internal consistency and stability of the measurement tool (Koo & Li, 2016), was assessed using Cronbach’s α coefficients. In this study, the total scale achieved a Cronbach’s α coefficient of 0.922. The three subscales had Cronbach’s α coefficients of 0.899, 0.960, and 0.954, respectively. Compared with the original scale (Cronbach’s α = 0.89) and its subscales (Cronbach’s α coefficients of 0.82, 0.81, and 0.76), the current scale demonstrated higher internal consistency. All Cronbach’s α coefficients for the dimensions exceeded 0.8, indicating good internal consistency. This indicates that the adjustments made during the translation and cultural adaptation process have enhanced the conceptual clarity and internal consistency of the dimensions. The half-point reliability was 0.874 > 0.7, indicating high internal consistency. The scale was re-administered to 30 nurses following a two-week interval, when the sample size is ≥30, the sampling distribution approaches normality, and the results are more stable with good reliability (Xu et al., 2024), the retest reliability was 0.886, and the reflection scale has good stability across time. Overall, the NCDNP-N has good reliability.

Effective management of patient disease progression has become a significant global concern. Timely and appropriate communication among healthcare providers regarding changes in a patient’s condition is crucial for improving patient outcomes and preventing adverse events. In this context, interprofessional collaboration is not only a working model but also a working philosophy that emphasizes equal participation, effective communication, and joint decision-making among different professions, as well as respect and trust, and focuses on collaborative efficiency (Sarmadi et al., 2025). The three dimensions of this scale correspond exactly to the failures of “communication” and “coordination” in the theory. This theoretical connection not only validates the structural validity of our scale but also strengthens its theoretical basis as an assessment tool for interprofessional collaboration. Moreover, the difficulties in night shift healthcare collaboration are not only a team collaboration issue but also a key link in the patient safety management system (Segura-García et al., 2023). The Chinese-language NCDNP-N scale effectively captures the challenges faced by nurses and doctors during night shifts, identifies obstacles to collaboration, and provides insights into the clinical environment specific to nighttime nursing. The scale’s items are concise, easily comprehensible, and culturally relevant. Completion of the entire scale takes 3 to 5 min, with a high rate of completion. The results of this study have certain guiding significance for future research and clinical practice on the effectiveness of night shift collaboration between nurses and doctors. It can help systematically identify weak links in collaboration, thereby enabling targeted improvement measures. We suggest that hospital administrators regularly (*e.g.*, quarterly) use this scale to assess the nighttime collaboration situation in order to detect problems in a timely manner. Through scale assessment, managers can clearly identify the main issues in nighttime collaboration, such as low communication efficiency, unclear responsibilities, or insufficient team cohesion. The scale results can be directly used to guide the design of training programs. For example, if the assessment reveals prominent communication issues, targeted communication skills training can be conducted; if unclear responsibilities are found, job responsibilities and processes can be optimized. This scale is not only suitable for one-time assessments but can also be integrated into the hospital’s continuous quality improvement system. Through regular assessment, feedback, and intervention, hospitals can gradually form a virtuous cycle of “assessment - feedback - improvement - reassessment,” continuously enhancing the quality of nighttime collaboration. Moreover, this tool reflects the interpersonal dynamics between physicians and nurses, the culture of relationships in night shift care communication holds great significance, as a positive interpersonal dynamic is highly beneficial for facilitating communication among night shift medical staff, which is crucial for mitigating workplace conflicts, enhancing medical cooperation, and ensuring patient safety (Hossny & Sabra, 2021). However, this scale currently assesses the challenges of night-shift collaboration between nurses and physicians solely from the nurses’ perspective. Given their distinct roles in patient care, nurses and physicians might perceive different difficulties and contributing factors related to night-shift collaboration. Therefore, future research should also incorporate the perspectives of physicians to comprehensively analyze the background factors influencing night-shift collaboration.

## Limitations

This study still exists some limitations. Firstly, this study employed convenience sampling and a cross-sectional design, which may have compromised the representativeness of the sample. Moreover, this study only selected nurses from three provinces in China, the sample had geographical limitations. And the participants engaged in self-reporting, which may have introduced bias into the results. Future studies should consider broadening the sample size to better validate the applicability of this scale among Chinese nurses. In addition, It is recommended that the scale be applied to other medical industries or rural hospitals in the future to better ensure patient safety.

## Conclusion

This research involved translating the NCDNP-N scale into Chinese and adapting it to the Chinese cultural context, and subsequently evaluated its psychometric properties. The findings indicated that the reliability and validity of the 10-item NCDNP-N scale both reached a relatively ideal state. Owing to its straightforward content and structure, the scale is appropriate for assessing the extent of nighttime collaboration difficulties between nurses and physicians in China. Moreover, it provides a basis for future intervention studies designed to improve the efficiency of night shift collaboration between these two professional groups.

##  Supplemental Information

10.7717/peerj.21097/supp-1 Supplemental Information 1 Exploratory Factor Analysis Data Raw data

10.7717/peerj.21097/supp-2 Supplemental Information 2 Confirmatory Factor Analysis Data Raw data.

10.7717/peerj.21097/supp-3 Supplemental Information 3 Codebook

## Additional Information and Declarations

### Data Availability

The following information was supplied regarding data availability: The raw data is available in the Supplemental Files.
