# Supplementary material for: Reliability and validity of the Chinese version of Nighttime Collaboration Difficulties between Nurses and Physicians for Nurses (NCDNP-N) scale
Source: PeerJ. 2026 May 20;14:e21097. doi: 10.7717/peerj.21097 (PMC13198198; doi:10.7717/peerj.21097)
Supplement: Supplemental Information 3 [file peerj-14-21097-s003.doc]

Codebook

| Score | Representative |
| --- | --- |
| 0 | Never |
| 1 | Seldom |
| 2 | Sometimes |
| 3 | Often |
| 4 | Always |
